# Development of sandwich dot-ELISA for specific detection of Ochratoxin A and its application on to contaminated cereal grains originating from India

**DOI:** 10.3389/fmicb.2015.00511

**Published:** 2015-05-26

**Authors:** M. Venkataramana, R. Rashmi, Siva R. Uppalapati, S. Chandranayaka, K. Balakrishna, M. Radhika, Vijai K. Gupta, H. V. Batra

**Affiliations:** ^1^Division of Toxicology and Immunology, DRDO-BU Center for Life Sciences, Bharathiar University, Coimbatore India; ^2^Microbiology Division, Defence Food Research Laboratory, Mysore India; ^3^Department of Studies in Biotechnology, University of Mysore Mysore, India; ^4^Discipline of Biochemistry, School of Natural Sciences, National University of Ireland Galway Galway, Ireland

**Keywords:** Ochratoxin A, ELISA, monoclonal antibodies, HPLC, cereal grains

## Abstract

In the present study, generation and characterization of a highly specific monoclonal antibody (mAb) against Ochratoxin A (OTA) was undertaken. The generated mAb was further used to develop a simple, fast, and sensitive sandwich dot-ELISA (s-dot ELISA) method for detection of OTA from contaminated food grain samples. The limit of detection (LOD) of the developed enzyme-linked immunosorbent assay (ELISA) method was determined as 5.0 ng/mL of OTA. Developed method was more specific toward OTA and no cross reactivity was observed with the other tested mycotoxins such as deoxynivalenol, fumonisin B1, or aflatoxin B1. To assess the utility and reliability of the developed method, several field samples of maize, wheat and rice (*n* = 195) collected from different geographical regions of southern Karnataka region of India were evaluated for the OTA occurrence. Seventy two out of 195 samples (19 maize, 38 wheat, and 15 rice) were found to be contaminated by OTA by s-dot ELISA. The assay results were further co-evaluated with conventional analytical high-performance liquid chromatography (HPLC) method. Results of the s-dot ELISA are in concordance with HPLC except for three samples that were negative for OTA presence by s-dot ELISA but found positive by HPLC. Although positive by HPLC, the amount of OTA in the three samples was found to be lesser than the accepted levels (>5 μg/kg) of OTA presence in cereals. Therefore, in conclusion, the developed s-dot ELISA is a better alternative for routine cereal based food and feed analysis in diagnostic labs to check the presence of OTA over existing conventional culture based, tedious analytical methods.

## Introduction

The incidence of microfungi and their secondary metabolites (mycotoxins) is a worldwide phenomenon affecting all major cereal crops. Though the presence and incidence of mycotoxins in cereals have been demonstrated since the early origins of organized crop cultivation and have impacted mankind, their effects have largely been ignored until the recent past 40 years. Due to the rapid rise in population growth and the ever-increasing need for food and feedstuffs, interest has been laid on tackling the factors influencing the crop production viz., pre-harvest predicaments (like bacterial and fungal diseases, insect and pest infestations etc.) and post-harvest problems (like fungal and bacterial metabolite contamination). The accidental consumption of mycotoxin contaminated food and feed stuffs can cause several acute and chronic diseases in human and animals (Zain, 2011) and such intoxications or ‘physiological abnormalities’ resulting from exposure to mycotoxins are termed as “Mycotoxicosis.” In general, mycotoxicosis is not pathognomonic; therefore, determining the cause of the specific condition or disease requires confirmation of the toxin(s) in a representative sample of the feed, food, tissue, or fluid (Richard et al., 1993). The potency and sturdiness of mycotoxins and the significant losses annually to the health, trade, economy and the marketing of foods and feeds have attracted worldwide attention toward undertaking active research on mycotoxin detection and analysis (Priyanka et al., 2014; Ramana et al., 2014).

Ochratoxin A (OTA; C_20_H_18_ClNO_6_, molecular weight = 403.82 g/mol) is one of the important and most potent mycotoxins produced by different species of *Penicillium* (*P. verrucosum, P. chrysogenum, and P. nordicum*) and *Aspergillus* (*A. ochraceus, A. melleus, A. sulphureus, Aspergillus section Nigri, A. carbonarius, A. awamori*; Bayman and Baker, 2006; Rashmi et al., 2013; Priyanka et al., 2014). The toxin is found mainly to contaminate cereal grains (wheat, corn, rye, barley) but it can also be found in rice, soybeans, coffee, cacao, beans, peas, peanuts, and dry fruits like figs, raisins, etc. (Kuruc et al., 2015; Palumbo et al., 2015). It is also present in beer (Nguyen and Ryu, 2014), wine and grape juice (Terra et al., 2013). OTA has been classified by the International Agency of Research in Cancer (IARC) as a carcinogen of 2B class (Muscarella et al., 2004). Its hepatotoxic, nephrotoxic, and teratogenic effects were well-documented (Mor et al., 2014; Mantle et al., 2015) and it is also involved in the Balkan endemic nephropathy (BEN) and in the Chronic Interstitial Nephropathy (Bayman and Baker, 2006).

Traditionally, OTA mycotoxin is detected by various analytical techniques of which thin layer chromatography (TLC) and high-performance liquid chromatography (HPLC) are the most commonly used techniques. These methods are rapid, sensitive and accurate and can be effectively used for definitive determination of OTA from various food and feedstuffs. With the advent of hybridoma technology, many monoclonal antibody (mAb) based detection platforms are being developed for assessment of mycotoxin levels in food and feedstuffs. Many researchers have developed immunology based assays like enzyme-linked immunosorbent assay (ELISA) for analyzing OTA contamination in cereals, dried fruits, coffee, cocoa, tea, beer, wine, and grape samples (Giesen et al., 2010; Zhang et al., 2011; Novo et al., 2013). Alternative methods like Fluorescence immunoassay (Zezza et al., 2009) and Aptamer-based assays (Liu et al., 2015; Rivas et al., 2015) are also developed for assessment of OTA. There are few reports on the occurrence of OTA in the Indian conditions mainly because the available analytical tools are neither user friendly nor economical for routine analysis. Therefore, in the present study, a rapid, reliable, and easy-to-perform sandwich dot-ELISA (s-dot ELISA) for the sensitive and specific detection of OTA was developed. To demonstrate the utility of the developed method, a total of 195 cereal grain samples from different regions of Southern Karnataka region of India were collected and processed for the analyzing the OTA contamination by s-dot ELISA and HPLC techniques.

## Materials and Methods

### Materials

Ochratoxin A, Ochratoxin B (OTB), deoxynivalenol (DON), aflatoxin B1 (AFB1), fumonisin (FB1), bovine serum albumin (BSA), ovalbumin (OVA), *N*-(3-dimethylaminopropyl)-*N*-ethylcarbodiimide hydrochloride (EDC), *N*-hydroxy succinimide (NHS), Freund’s complete adjuvant (FCA), Freund’s incomplete adjuvant (FIA), streptomycin, penicillin, fetal bovine serum (FBS), polyethylene glycol 2000 (PEG 2000), 3,3,5,5-tetramethylbenzidine (TMB), diaminobenzidine (DAB), and myeloma cells (Sp2/0-Ag14) were acquired from Sigma-Aldrich (St. Louis, MO, USA). RPMI1640 culture medium was acquired from Gibco (Carlsbad, CA, USA). Goat anti-mouse IgG horseradish peroxidase (HRP) was acquired from (Sigma, USA). The working buffer utilized incorporated 0.1 M PBS, pH 7.4 (0.1 M phosphate support, 0.138 M NaCl, 0.0027 M KCl), and PBS with Tween 20 (PBST; 0.01 M PBS, 0.05% Tween 20). IsoStrip mouse monoclonal immunizer isotyping kit was acquired from Sigma-Aldrich (Sigma-Aldrich, USA). Unless otherwise specified, all expository evaluation reagents were gotten from Merck, Mumbai, India. Experimental animals were acquired from Defence Food Research Laboratory (DFRL), India. ELISA reader (Bio-Rad, Hercules, CA, USA) was utilized.

### Safety Note

Ochratoxins are profoundly cancer-causing and ought to be taken care of with compelling consideration. OTA contaminated material is always purified with a liquid solution of sodium hypochlorite (5%).

### Fungi

All the fungi used in the study were listed in **Table 1**.

**Table 1:** Standard cultures and fungal isolates used in present study.

Name	Code	Source
**OTA positive Aspergilli (*n* = 25)**		
*Aspergillus carbonarious* ITCC 2005	A1	ITCC^∗^, India
*A. ochracious* ITCC 1456	A2	ITCC, India
*A. ochracious* ITCC 3167	A3	ITCC, India
*A. ochracious* ITCC 2454	A4	ITCC, India
*A. ochracious* MTCC 1810	A5	MTCC^¥^, India
*A. ochracious* DFR_AO1-DFR_AO10	A6–A15	DFRL^§^, India
*A. carbonarious* DFR_AC1-DFR_AC10	A16–A25	DFRL, India
**OTA negative Aspergilli (*n* = 15)**		
*A. flavus* ATCC 46283	A26	ATCC^†^, USA
*A. flavus* NCIM 152	A27	NCIM^‡^, India
*A. flavus* NCIM 645	A28	NCIM, India
*A. flavus* NCIM 650	A29	NCIM, India
*A. flavus* MTCC 2798	A30	MTCC, India
*A. parasiticus* MTCC 2797	A31	MTCC, India
*A. flavus* DFR_AF1-DFR_AF9	A32–A40	DFRL, India
**OTA positive Penicillia (*n* = 32)**		
*Penicillium verrucosum* ITCC 2156	P1	ITCC, India
*P. verrucosum* ITCC 2986	P2	ITCC, India
*P. verrucosum* MTCC 1758	P3	MTCC, India
*P. viridicatum* MTCC 2007	P4	MTCC, India
*P. verrucosum* DFR_PVer1-DFR_PVer15	P5–P19	DFRL, India
*P. verrucosum* DFR_PVir1-DFR_PVir13	P20–P32	DFRL, India
**OTA negative Penicillia (*n* = 20)**		
*P. citrinum* DFR_PCit1-DFR_PCit10	P33–P42	DFRL, India
*P. chrysogenum* DFR_PChr1-DFR_PChr5	P43–P47	DFRL, India
*P. hirsutum* DFR_PH1-DFR_PH5	P48–P52	DFRL, India
**Other OTA negative fungi (*n* = 8)**		
*Fusarium graminearum* MTCC 2089	F1	MTCC, India
*Fusarium verticillioides* MTCC 3693	F2	MTCC, India
*Fusarium sporotrichoides* MTCC 2081	F3	MTCC, India
*Penicillium chrysogenum* MTCC 6479	F4	MTCC, India
*Fusarium moniliforme* MTCC 156	F5	MTCC, India
*F. proliferatum* MTCC 286	F6	MTCC, India
*F. culmorum* ITCC 149	F7	ITCC, India
*F. solani* ITCC 3359	F8	ITCC, India

^∗^Indian Type Culture Collection (ITCC); ^¥^Microbial Type Culture Collection (MTCC); ^§^ Defence Food Research Laboratory; ^†^American Type Culture Collection; ^‡^National Collection of Industrial Microorganisms.

### Conjugation of OTA to BSA

Ochratoxin A was coupled to BSA in the presence of EDC and NHS as described previously (Yu et al., 2005). Five milligrams of OTA was dissolved in 0.5 mL dimethyl sulfoxide (DMSO) that contained 6 mg NHS and 8 mg EDC. The reaction was maintained at room temperature for 2 h in the dark and then at 4°C overnight. The solution was added to 10 mg imject BSA solution (Life Technologies, Bengaluru) drop-wise and then dissolved in 2 mL 0.1 M carbonate buffer (pH 9.6), and maintained at room temperature for 2 h. Following the reaction, the mixture was dialyzed against 10 mM PBS (pH 7.4), with the buffer replenished six times over 72 h.

### Conjugation of OTA to OVA

Ochratoxin-OVA conjugate was prepared by coupling OTA to OVA in the presence of a water-soluble carbodiimide (Chu et al., 1976) and used as solid-phase antigen for the indirect ELISA. In a typical reaction, 0.5 mg of OTA in 0.2 mL of conjugation buffer was mixed with 2.5 mg of imject OVA (Life Technologies, Bengaluru) followed by 1 mg of EDC was added to the mixture with constant stirring. After the coupling reaction was carried out at 25°C for 2 h, the mixture was dialyzed against PBS for 72 h and then lyophilized for storage.

### TNBS Assay

Different ratios of protein–hapten conjugates were characterized by determining the available groups of surface lysine present in carrier proteins before and after conjugation. This was accomplished by using 2,4,6-trinitrobenzene-1-sulfonic acid (TNBS) reagent. The amount of amino groups present in the carrier protein before and after coupling with carboxylated hapten was directly quantitated with a UV/vis spectrophotometer at 335 nm (Sashidhar et al., 1994). Different conjugates were prepared at a concentration of 1 mg/mL and were reacted with 0.1% TNBS solution under alkaline conditions to determine the percentage of NH_2_ groups used during conjugation in different conjugates. An amount of 200 μl of conjugate solution was taken and mixed with 200 μl of 4% NaHCO_3_ solution. An amount of 200 μl of 0.1% TNBS solution was added to the mixture and incubated for 1 h at 37°C. The OD of the solution was read at 335 nm. The amount of NH_2_ groups used during conjugation of OTA to the BSA/OVA molecules was determined from the difference between the OD of the control and the conjugate.

### Immunization of OTA-BSA

Female BALB/c mice were obtained from the animal facility center, Defence Food Research Laboratory (DFRL), Mysore. Initially, mice were housed in groups of three per cage with feed and tap water. Their general state of health was assessed daily and body weights were recorded weekly. The mice were maintained and used in accordance with the recommendations of the committee for the purpose of control and supervision of experiments on animals. Immunization started at an age of 7 weeks. Fifty micrograms of OTA-BSA was emulsified with an equal volume of FCA, and 6–8 weeks-old female BALB/c mice were immunized subcutaneously at day 0 and subsequent doses were given with FIA at days 14, 28, and 44. The mice with high antisera against OTA were finally boosted intraperitoneally with 50 μg crude conjugate 3 days before the fusion. Blood samples (approximately 0.1 ml blood from the plexus retrobulbaris) were collected at defined intervals. To minimize the risk of blindness, each mouse served as blood donor only four times. Sera were prepared by centrifugation (2000 *g*, 10 min) of the coagulated blood and stored as aliquots at -20°C until further use. A separate group of mice (*n* = 3) received sham immunizations with PBS and adjuvants and the sera from this group were collected to use as negative control.

New Zealand White rabbits (6 weeks-old, female) were immunized intra-dermally with OTA-BSA conjugate. Primary immunization of each animal was done with 50 μg of OTA-BSA in FCA. Three boosts of 50 μg protein in FIA followed on days 14, 28, and 35.

### Hybridoma

Hybridoma was carried out as per Köhler and Milstein (1975) with minor modifications; the spleen of the immunized mouse was aseptically removed and fused with SP2/0-Ag14 cells. SP2/0-Ag14 cells were cultured in RPMI 1640 with 100 mg/mL streptomycin and 100 U/mL penicillin, in a CO_2_ incubator set at 37°C and 5% CO_2_ concentration. Mouse lymphocytes (10^8^ cells) were mixed with the SP2/0-Ag14 myeloma cells at the ratio of 5–10:1 and centrifuged at 2000 rpm for 5 min. One milliliter volume of PEG 2000 was warmed to 37°C and dropped onto the cells pellet within 1 min. After adding 30 mL culture medium without FBS, the cells were incubated for 10 min at 37°C. The fused cells were centrifuged for 10 min and resuspended in 20 mL complete medium with HAT. Cells were seeded into 96 well-culture plates with feeder layers of mouse peritoneal macrophages, and extended in a CO_2_ incubator set at 37°C and 5% CO_2_ concentration. After 5 days, complete medium with hypoxanthine, aminopterin, and thymidine (HAT) was added to about half the volume of the well. At 50% confluence, the antibody-secreting hybridoma cells were detected by indirect ELISA. Positive hybridoma cells were grown in hypoxanthine and thymidine (HT) medium. The hybridoma cells that produced the most sensitive supernatant fluid were cloned using the limited dilution and extension method. The cell supernatant fluid was subjected to indirect ELISA again 10 days after fusion. The positive hybridoma cells were subsequently extended by limiting dilution. Sub-cloning was repeated three times to obtain positive mAb producing cells, then amplified and frozen in liquid nitrogen. The anti-OTA mAb was produced by the mouse ascites method (Cho et al., 2005).

### Antibody Purification and Analysis

The four OTA-mAbs generated and the rabbit hyperimmune polysera were purified by the ammonium sulfate precipitation method and protein A column affinity chromatography. Different fractions obtained during the purification of IgG were subjected to gel electrophoresis to check for purity. Using a mini-PROTEAN II Electrophoresis Cell (Bio-Rad, India), samples in lysis sample buffer (containing 25% glycerol, 2% SDS, 5% 2-mercaptoethanol, 0.01% bromophenol blue and 62.5 mM Tris-HCl, pH 6.8) were applied to a 12% acrylamide-bis gel (Bio-Rad, India) and proteins were separated by SDS-PAGE in Tris/Glycine/SDS running buffer, pH 8.3 [containing 25 mM Tris-base, 192 mM glycine, and 0.1% (w/v) SDS] for 45 min with a constant voltage of 200 V. Proteins were stained for 30 min with 0.1% Coomassie blue R-250 in 40% methanol (MeOH) and 10% acetic acid (HOAc); subsequently the gel was destained with several changes of 40% MeOH/10% HOAc for 1–3 h.

### Isotyping of the mAb

Isotyping test was carried out with IsoStrip mouse monoclonal antibody Isotyping Kit (Sigma-Aldrich, Bengaluru, India), according to the manufacturer’s instructions.

### Indirect ELISA

The antibody reactivity of immunized rabbit sera was measured by indirect plate-ELISA. OTA-OVA (0.5 μg/well) in 0.05 M carbonate/bicarbonate buffer was added to the culture wells and kept at 4°C overnight. One percent gelatin in PBS solution was used to block the wells for 2 h at 37°C, and 100 μL diluted (1:1000) antisera was added and left to bind for 1 h at 37°C. Approximately 100 μL goat anti-rabbit IgG-HRP (1:1000 in PBST) was added to each well and incubated for 1 h at 37°C. Between each of the above steps, the wells were washed three times with PBST. Finally, the wells were incubated with TMB/H_2_O_2_ substrate for 15 min at 37°C. The absorbance at 450 nm was measured after the reaction was stopped with 50 μL of 0.2 M H_2_SO_4_.

### Indirect dot-ELISA

Ten μl aliquots of individual toxin conjugates at a concentration of 100 μg/ml were manually dotted onto nitrocellulose membrane (Pall, India). Sterile PBS (pH 7.4) served as negative control. The NC membrane was air dried, blocked by placing into 5% skim milk in PBS (pH 7.4), incubated at 45°C for 30 min, and washed with three changes of 1× PBST (PBS + 0.5% Tween 20). The membrane was then probed with OTA1 mAb for 30 min at room temperature. After the incubation, the membrane was washed with PBST as described above and then incubated for 30 min at room temperature with HRP conjugated rabbit anti-mouse Igs (Dako, Denmark). After 30 min, the membrane was washed with PBST three times, immersed in the substrate solution (TMB + 0.4% H_2_O_2_) for color development for 1 min, washed with distilled water, and air dried.

### Specificity of Antibodies

Specificity of the polyclonal antisera and mAb was checked by using series of mycotoxin conjugates (DON-BSA, FB1-OVA, AFB1-BSA) by indirect ELISA as mentioned earlier.

### Sandwich-dot ELISA

The nitrocellulose (NC) membrane strips were divided into squares 1.0 cm × 1.0 cm with a hard lead pencil, and 20 μL aliquots of a range of rabbit polyclonal IgG (1–5 mg/ml) against OTA-BSA diluted in PBS were dotted on separate squares. The strips were allowed to dry and then the remaining unbound sites were blocked with a solution of 5% skim milk in PBS. After washing with PBST, the strips were cut into the squares and placed in 24 well microtitre plate. Two hundred μL to 1 ml of suspected sample containing OTA was added into each well and the plate was incubated at 37°C for 1 h. Each assay always had OTA positive and OTA negative control antigens. The NC squares were washed and then incubated at 37°C for 1 h with 500 μL of the 0.5 μg/ml mouse monoclonal IgG against OTA. After washing with PBST, the plates were incubated at 37°C with 1:5000 diluted HRP conjugated rabbit anti-mouse Igs (Dako, Denmark). After 45 min, the membrane was washed with PBST three times, immersed in the substrate solution (DAB + 0.4% H_2_O_2_) for color development for 1 min, washed with distilled water and air dried.

### Specificity of s-dot ELISA

Cross-reactivity was checked with s-dot ELISA using different mycotoxins viz. FB1, DON, AFB1, and OTB.

### Sensitivity of s-dot ELISA by Dilution Method

Sensitivity of developed s-dot ELISA was estimated using OTA dilutions. Standard OTA was serially diluted ranging from 250 to 1 ng/ml and 1 ml of each dilution was used in the s-dot ELISA.

### Extraction of OTA from Fungal Cultures

Single spore cultures of fungal strains in **Table 1** were inoculated onto Potato dextrose broth (Himedia, Mumbai). After 6 days of incubation at 28°C, mycelium was filtered and spore suspension was collected and inoculated into CYA medium for toxin production for 10 days of incubation at 30°C and 150 rpm on a rotary shaker. Mycelium was filtered by using sterile filter paper and 25 mL of methanol was added to 25 mL of filtrate, shaken well and extracted with 25 mL of ethyl acetate. After extraction, ethyl acetate was completely evaporated by rotary evaporation and the mycotoxins were resuspended in 0.5 mL of methanol. These samples are used directly for s-dot ELISA.

### Cereal Samples and OTA Extraction

A total of 195 cereal samples (55 wheat, 80 maize, and 60 rice) collected from different storage houses and farming fields of southern Karnataka region were subjected to HPLC and s-dot ELISA for analyzing the presence of OTA mycotoxin. OTA from cereal samples was extracted according to method described in Solfrizzo et al. (1998), with minor modifications. Briefly, cereal samples were finely ground and 50 g of each sample was processed for toxin extraction. Samples were mixed with 30 ml chloroform and 2.5 ml 0.1 M phosphoric acid by shaking for 30 min on a rotary shaker. The extract was filtered through a fluted Whatman number 1 filter paper. A 15 ml volume of filtrate was collected and evaporated using rotary evaporator. The residue was reconstituted in 2 ml of acetonitrile–water–acetic acid (41:58:1, v/v) by vortexing for 1 min. The reconstituted extract was defatted with 1 ml n-hexane by vortexing for another 1 min and centrifugation at 10,000 rpm for 10 min. The lower phase was collected and filtered through 0.45 μm syringe filter. This sample is directly used for s-dot ELISA. For HPLC analysis, the sample was subjected to clean-up. Specific immune-affinity columns (VICAM, Watertown, MA, USA) were used for clean-up of OTA following the manufacturer’s protocol. Elutions were performed in 3 ml of methanol. Standard OTA solution (Sigma-Aldrich, Bengaluru, India) was prepared according to supplier’s instructions.

### High-Performance Liquid Chromatography

Ten microlitres of toxin extracts were injected into the RP-C18 column (Jasco, Great Dunmow, Essex, UK) with dimensions of 3 μm and 250 mm × 46 mm for HPLC analysis. For the OTA analyte, a methanol/water solution in the ratio 7:3 v/v (isocratic solution) was used as mobile phase. A Jasco HPLC system with fluorescence detector and wavelength settings of excitation 365 nm and emission 455 nm with a flow rate of 0.8 ml/min was used for OTA determination.

### Statistical Analysis

The data were presented as mean ± SD. All graphical illustrations were constructed either by GraphPad Prism5 software or in Microsoft Excel sheet. Student’s *t*-test was used for all statistical comparisons. Significance (*P*) value summary: ^∗^*P* ≤ 0.05; ^∗∗^*P* ≤ 0.01; ^∗∗∗^*P* ≤ 0.001; ^∗∗∗∗^*P* ≤ 0.0001.

## Results

### Analysis of OTA-Protein Conjugates

**FIGURE 1:**
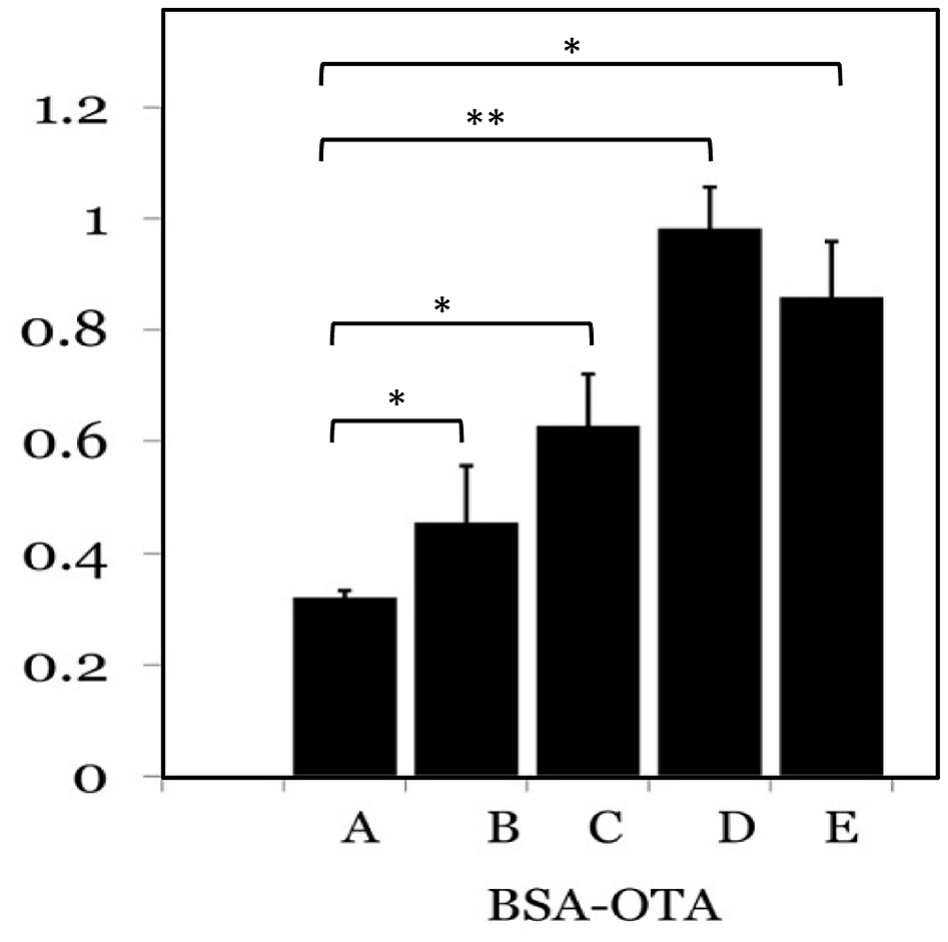
**Determination of antigenicity of protein: OTA conjugates.** Different protein to OTA ratios were used as antigens and probed with specific sera from mice. A-1:5; B-1:10; C-1:25; D-1:50; E-1:100 protein to OTA ratio. ^∗^*P* ≤ 0.05; ^∗∗^*P* ≤ 0.01.

Ochratoxin A-BSA conjugate was prepared for the immunization animals and OTA-OVA conjugate was made for the indirect ELISA as the coating antigen both the conjugations were accomplished by a carbodiimide method. The strategy of utilizing distinctive conjugate proteins for the immunization of animals and the coating of ELISA plates was useful for successful screening for OTA specific mAbs. The covalent attachment of carboxylic acid haptens to protein (BSA/OVA) via available amino groups (30–35 L-lysines in BSA and 20 in OVA) was confirmed by reacting all the prepared conjugates with TNBS reagent. The number of amino groups present in the carrier protein before and after conjugation was quantitated and shown in **Table 2**. The gradual decrease in the available free lysine on protein after reaction with different molar ratios of OTA confirms the increase in conjugation with increasing OTA protein molar ratio. The OTA-OVA conjugate to be used for ELISA plate coating was found to be conjugated maximum at a OVA:OTA molar ratio of 1:100. The OTA-BSA conjugate was used to immunize BALB/c mice subcutaneously by injecting 25 μg each BSA:OTA conjugate ratio emulsified with FCA. Following this, three secondary boosters of same dose in incomplete Freund’s adjuvant at intervals of 14 days each was injected into mice. The mice were bled on the third day after the final boost, and the antibody titre was determined by indirect ELISA using OTA-OVA antigen. The ratio of 1:50 BSA-OTA conjugate, in spite of its incomplete saturation generated high titres of specific antibodies (**Figure 1**).

**Table 2:** Determination of OTA density on BSA and OVA conjugates using chemical TNBS method.

Protein to OTA ratio	BSA-OTA conjugate		OVA-OTA conjugate	
	Percent NH2 used	No. of NH2 used	Percent NH2 used	No. of NH2 used
1:0	0	0	0	0
1:5	5	3.56 (4)	2	2.26 (2)
1:10	7	4.92 (5)	5	5.11 (5)
1:25	15	12.12 (12)	9	10.64 (11)
1:50	21	16.32 (16)	11	15.35 (15)
1:100	25	18.24 (18)	15	17.92 (18)

### Production of Antibody

**FIGURE 2:**
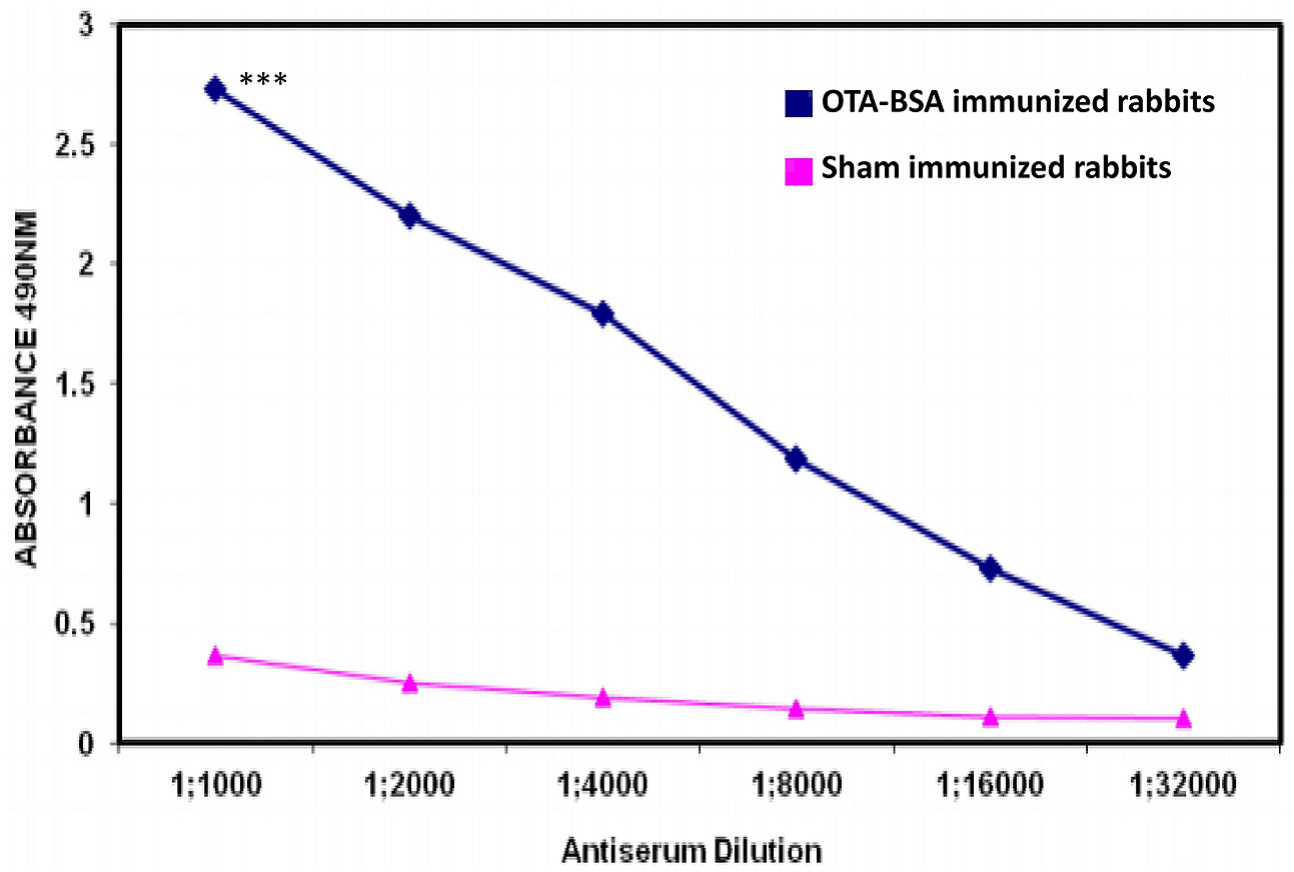
**Antibody titres induced by immunization of OTA-BSA conjugate to New Zealand White rabbits.** ^∗∗∗^*P* ≤ 0.001.

New Zealand White rabbits and BALB/c mice immunized with 50 μg/dose of OTA-BSA conjugate following the immunization regimens mentioned in Section “Materials and Methods” achieved antibody titres of 1:32,000 (**Figure 2**) and 1:64,000, respectively, by 4 weeks post to the initiation of immunization. As the titre estimation was done by indirect ELISA using 0.5 μg/well OTA-OVA coated 96-well-plates, the observed titres would be considerably represented by anti-OTA antibodies rather than from BSA. Hyperimmunized mouse splenocytes were used to generate hybridomas and four candidate anti-OTA mAb producing clones that were highly reactive were selected for further investigation. Indirect ELISA method, where plates were coated with BSA protein alone and OTA-OVA conjugate, was used to screen the positive clones and to assess the strength of binding against OTA, respectively. Out of four clones that were characterized, clone termed OTA1 exhibited higher reactivity toward OTA-OVA, and this clone was expanded for further experiments.

### Characterization of OTA1 Monoclonal Antibody

#### Isotyping

Isotyping of the clone OTA1 was carried out and the mAb was identified as IgG2a subtype, with the light chain belonging to the kappa configuration.

#### Specificity of OTA1 mAb

**FIGURE 3:**
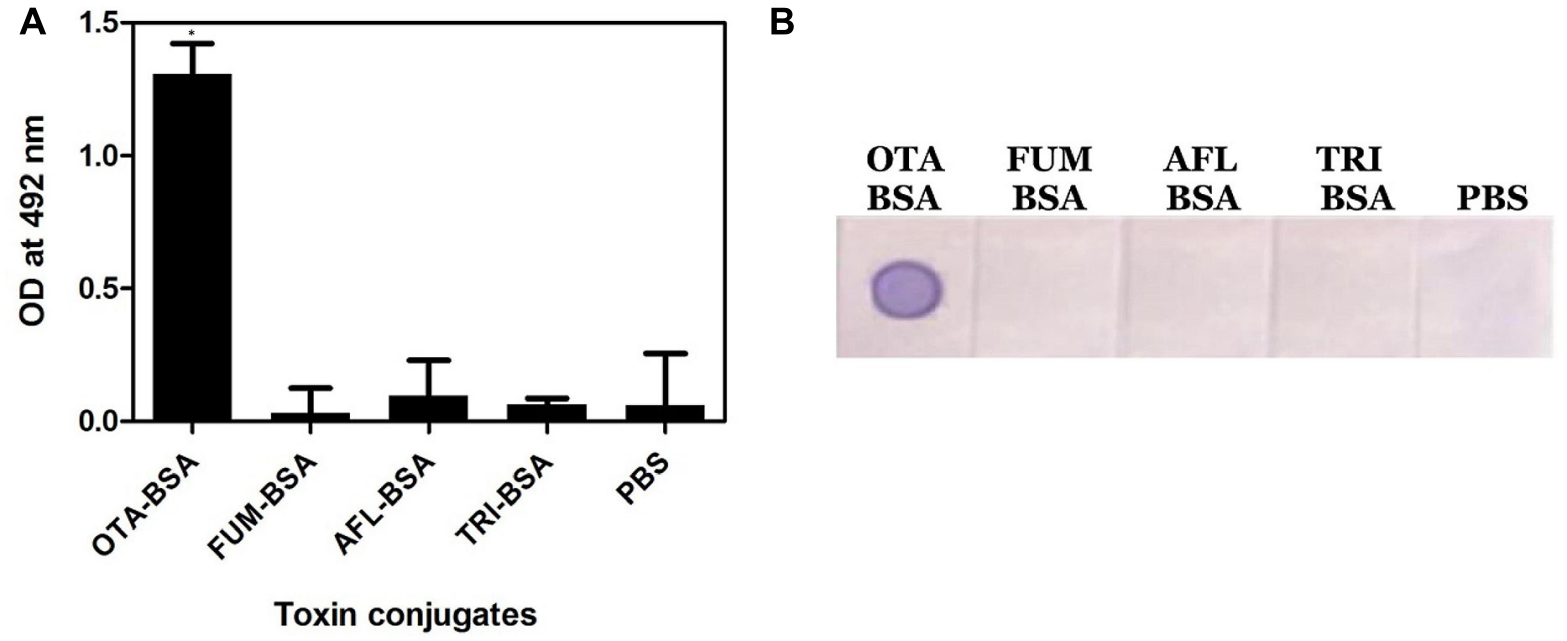
**Specificity of anti-OTA mAb. (A)** Indirect plate ELISA, **(B)** dot ELISA. ^∗^*P* ≤ 0.05.

Specificity of the anti-OTA mAb was assessed by performing Indirect plate and dot-ELISA on different mycotoxin conjugates (**Figure 3**). In both the cases, the OTA1 mAb reacted specifically with OTA conjugate alone. No background was observed.

### Standardization of Sandwich dot ELISA

The s-dot ELISA format developed in the present study resembles the traditional dot-ELISA except for an initial step involving capture antibody coating. The protocol is mentioned in detail in methodology section. For standardization, both anti-OTA mAb and anti-OTA polyclonal rabbit sera were tried upon as capture and revealing antibodies. After testing the format for reproducibility and reliability, the optimized protocol utilized 3 mg/ml anti-OTA polyclonal rabbit sera as coating antibody and anti-OTA mAb was utilized as revealing antibody. Also, the dot size was standardized to contain 20 μl volume of capture antibody in 1 sq.cm area of nitrocellulose membrane.

### Specificity and Sensitivity of Sandwich dot ELISA

**FIGURE 4:**
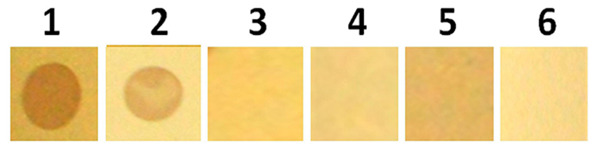
**Specificity of s-dot ELISA. 1-OTA; 2-OTB; 3-AFB1; 4-FB1; 5-DON; 6-PBS (negative control)**.

**FIGURE 5:**
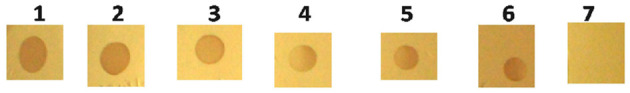
**Sensitivity of s-dot ELISA.** 1: 250 ng/ml of OTA; 2: 100 ng/ml of OTA; 3: 50 ng/ml of OTA; 4: 25 ng/ml of OTA; 5: 10 ng/ml of OTA; 6: 5 ng/ml of OTA; 7: 1 ng/ml.

When assessed for cross-reactivity against other major mycotoxins, the developed s-dot ELISA method was specific to OTA, although, it exhibited weak cross reaction with OTB (**Figure 4**). OTA and OTB are structurally related mycotoxins, where OTA has just an extra chlorine atom. Although it is not uncommon that anti-OTA antibodies react with OTB, the weak reaction indicates that either the capture antibody or revealing antibody might bind to OTB. When assessed using OTB-BSA conjugate in dot-ELISA format, anti-OTA rabbit polysera reacted moderately with OTB whereas mAb reacted weakly (data not shown). On the other hand, no cross-reactivity was observed with other major mycotoxins such as AFB1, FB1, and DON. Sensitivity of the assay showed as a minimum limit of 5 ng/ml of standard OTA in dot ELISA (**Figure 5**).

### Detection of OTA from Crude Fungal Exudates

**FIGURE 6:**
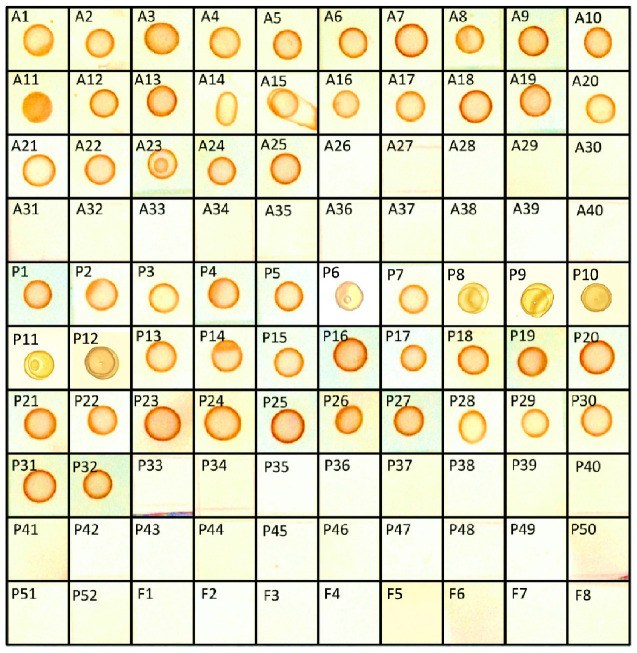
**Detection of OTA positive fungal cultures by s-dot ELISA**.

Crude mycotoxins were extracted from all the 40 *Aspergillus*, 52 *Penicillium,* and eight other fungal isolates listed in the **Table 1** and were subjected to s-dot ELISA. All the 25 *Aspergillus* and 32 *Penicillium* strains that were previously proven to produce OTA gave positive results by s-dot ELISA (**Figure 6**). None of the OTA negative strains showed positive reaction by s-dot ELISA.

### Evaluation of OTA from Field Samples

**FIGURE 7:**
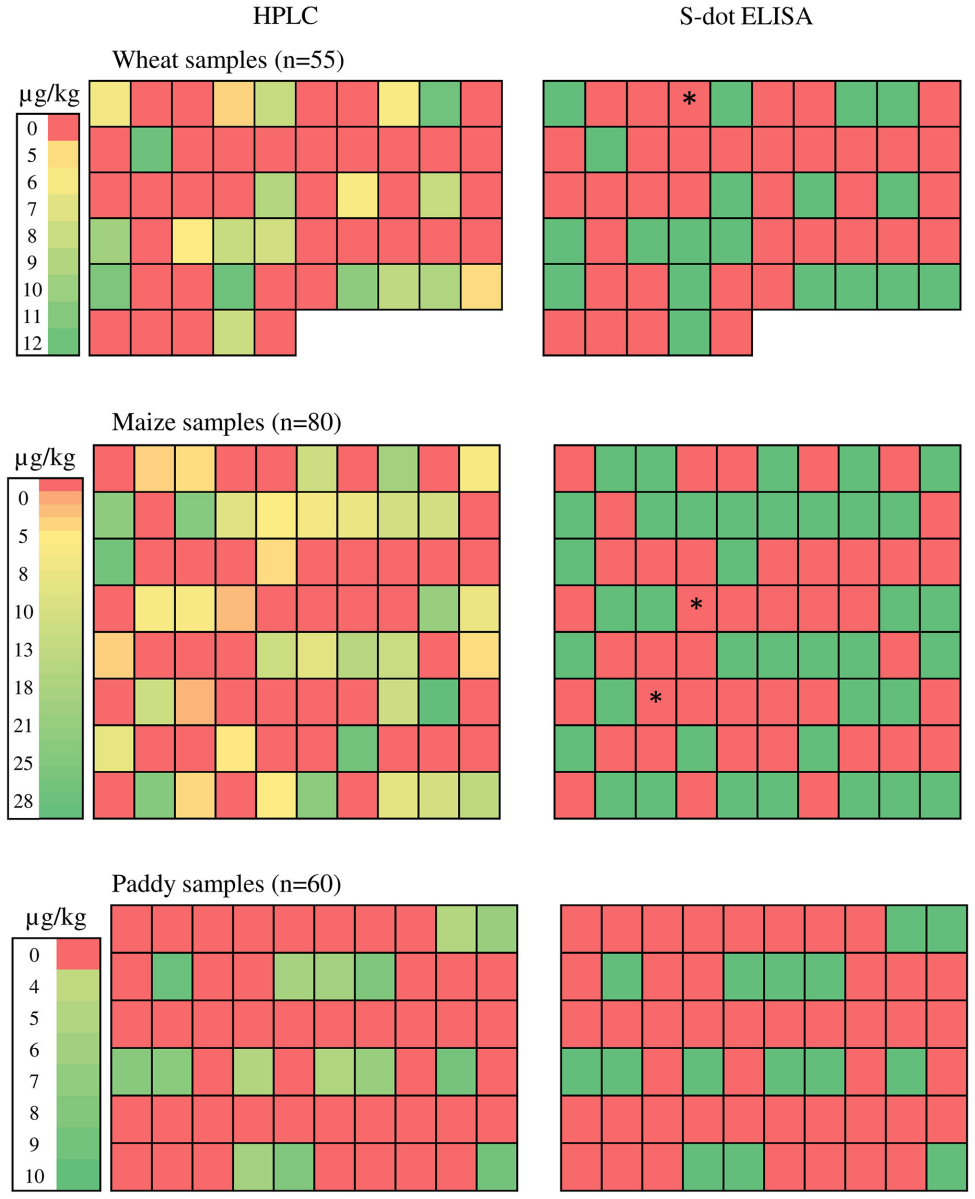
**Ochratoxin A estimation from natural cereal samples.** ^∗^ represents W4 (wheat), M44 and M63 (Maize) samples with OTA contamination within range of 3.3–4.6 μg Kg^-1^ and were found positive for HPLC but showed negative by s-dot ELISA.

A total of 195 cereal [wheat (55), maize (80), and rice (60)], samples were analyzed for occurrence of OTA by the s-dot ELISA method developed in this study as well as HPLC method. Twenty out of 55 wheat samples contained OTA in a range of 4.604 – 12.1 μg Kg^-1^, with a mean level of 8.61 μg Kg^-1^. Forty out of 80 maize samples were found to be OTA positive, ranging from 3.3 to 27.021 μg Kg^-1^ with a mean level of 13.67 μg Kg^-1^. Fifteen out of 60 rice samples were positive for OTA, ranging from 4.9 to 9.67 μg Kg^-1^, with a mean level of 6.65 μg Kg^-1^ (**Figure 7**). W4 (wheat), M44 and M63 (Maize) samples (represented as ^∗^ in **Figure 7**) were contaminated with OTA within range of 3.3–4.6 μg Kg^-1^ and were found positive for HPLC. But these samples were found negative by s-dot ELISA probably due to the limited yield of the OTA extraction protocol.

## Discussion

Detection of OTA from various food matrices has been a target for many researchers and as a result of incessant and strenuous efforts; many formats of detection were developed. The most common methods applied for detection of OTA are TLC and HPLC. HPLC is the most sensitive technique ever developed for analysis of all the mycotoxins, but it requires cumbersome clean-up processes and skilled manpower, besides costly reagents and instruments. On the other hand, TLC although cheaper and easy to perform, requires extensive sample preparation and is not a sensitive method. Hence, immunoassays are being developed which are reliable, fast, sensitive and easy to perform (Ghali et al., 2008; Uchigashima et al., 2012; Vidal et al., 2012). In general, immunoassays for OTA detection utilize either specific polyclonal or mAbs in sandwich indirect ELISA format. Immunoassays are favored over TLC and HPLC because of their non-requirement of extensive sample clean-up process and low cost. But, the major drawbacks associated with these sandwich immunoassays are necessity of spectrophotometer to analyze the results and the low volume of sample to be analyzed. To address these problems, a mAb based s-dot ELISA format with all the beneficial properties of conventional immunoassays and none of aforementioned drawbacks is developed in the present study. For instance, as the present assay is a ‘dot’ ELISA, the assay is done on a nitrocellulose membrane where the result appears as a dark dot on a white background which can be visualized without any equipment. Three important features of the present dot ELISA format render its utility in routine food analysis; easy toxin extraction protocol, high sensitivity and low cost. The toxin extraction protocol for the present assay is optimized without any use of extensive sample clean-up procedures before the assay. The amount of toxin extract to be analyzed in the present dot ELISA can be as much as 1–5 ml in contrast to 100–200 μl in case of plate ELISA. This feature favors the sensitivity aspect of dot ELISA which was found to be as less as 5 ng/ml of OTA. Coupled with an easy toxin extraction protocol without any clean-up and high sensitivity, the assay requires a minimal time of 4–5 h and the cost involved in the whole procedure is very low. The only disadvantage with the present assay is that the dot ELISA format cannot be used for quantitative detection of OTA.

Development of immunoassay for mycotoxin detection requires addressing three major aspects for improving the limit of detection (LOD); immunogenicity of hapten–protein conjugate, avidity of mAb, and mycotoxin extraction protocol. Haptens are usually non-immunogenic inactive compounds and hence do not elicit an immune response on their own unless coupled with some macromolecules such as proteins. The most important aspect to be considered during specific antibody generation against a hapten is the linking of hapten to carrier protein. The conjugation method and the functional group of the hapten determine the immunogenicity of the hapten–protein complex. Generally, conjugation is mediated via amine, carboxylic acid, hydroxyl or sulphahydryl groups of hapten and the protein. Choice of carrier protein is also important in generation of specific antibodies for application in immunoassays. The most frequently used carrier proteins are BSA, OVA, conalbumin (CONA), thyroglobulin (TG), immunoglobulin (Ig), fibrinogen, or keyhole limpet hemocyanin (KLH). The choice of carrier protein depends on the solubility of the hapten–protein complex; for instance, complexes of serum proteins generally are soluble in pH 5.5, whereas complexes of KLH, γ-globulins etc. do not. Another major feature to be considered during hapten-carrier protein conjugation is the balance between hapten density on carrier protein and specificity of antibodies produced. The higher density of hapten on carrier protein increases the antigenicity of the hapten but on the other hand, high degree of substitution beyond a threshold may adversely affect the avidity and the specificity of immune response. In the present study, the number of hapten molecules per protein molecule was optimized by taking different hapten:carrier molar ratios and the ratio of 50:1 (OTA:BSA) was found to be optimal conjugation ratio for specific antibody generation.

The second aspect under consideration is the avidity of mAb against OTA hapten. Avidity is a measure of overall strength of antibody-antigen complex that defines the specificity and sensitivity of the antibody. Three parameters define the avidity of a mAb; affinity of the antibody to epitope, structure of the epitope and valency of antibody (Hudson and Kortt, 1999). Following a stringent immunization and Hybridoma screening protocol, the OTA-mAb in the present study is developed. Generally, splenocytes to be used in hybridoma technology will be extracted from hyperimmunized mice that have high titres of specific antibodies (Köhler et al., 1978). The protocol to determine the antibody titres is indirect plate ELISA where each well is coated with antigen at a concentration of at least 1 μg. In the present study, for all the plate ELISAs, the antigen (OTA-BSA conjugate) is used at a concentration of 0.5 μg/well and the final acceptable titre was five times the negative value where sham immunized mice sera is used. These two modifications in the current study facilitated the isolation of a specific mAb with highest affinity against OTA. Also, the OTA mAb is found to be IgG2 isotype indicating it to be a bivalent antibody. Previously many have developed anti-OTA mAbs with varied valencies like decavalent IgM (Candlish et al., 1986), bivalent IgG1 (Liu et al., 2008), and bivalent IgG2a (Cho et al., 2005). Although, IgG subclasses do not affect the avidity, it greatly affects the protective effect of the antibody. For instance, the length of the hinge region between the Fab arms and the two carboxy-terminal domains CH2 and CH3 of both heavy chains of IgG2 isotype is shorter than any other isotype (Canfield and Morrison, 1991). This restricts its flexibility rendering it to be a rigid antibody with extreme low affinity toward Fc receptor on phagocytic cells. The OTA1 mAb in the current study has high affinity toward OTA but being IgG2 subtype may have limited application in protective studies. To overcome this problem, in future, class switching experiments can be undertaken to render additional features in neutralization of OTA.

The third important aspect to be taken into account during development of an immunoassay for detection of mycotoxins is the mycotoxin extraction protocol. Many factors influence the binding of mAb to OTA in the immunoassays. As Visconti et al. (1999) pointed out, some of the metabolites of food commodities like anthocyanins and pigments of wine etc. can interfere with OTA binding to antibodies, giving false positive reactions. To overcome this problem, previous researchers have utilized immuno-affinity columns for clean-up of sample, but this procedure adds up to the cost and time of the assay (Varga and Kozakiewicz, 2006). The current s-dot ELISA format is developed to be employed in assessing OTA chiefly from cereal crops and when the OTA1 mAb is tested for cross reactivity with the crude methanolic and water extracts of sterile wheat, paddy, maize and sorghum cereals, no reactivity was observed. Hence, in the present study, the OTA extraction protocol does not use any immuno-affinity columns. To improve the sensitivity of the assay, we also employed organic solvent extraction of OTA followed by evaporation (Flajs et al., 2009). Using this modified protocol, the yield of OTA extracted from cereal samples increased to a great extent. Approximately, OTA from a 50 g of sample is finally extracted to a volume of 5 ml following the current protocol. This is very important because the maximum level of OTA contamination in raw cereals as mentioned in Codex Alimentarius Standard is 4–5 μg kg^-1^ sample. Following the present protocol, a 50 g of cereal sample contaminated with 5 μg kg^-1^ sample can ideally yield 5 ml extract of 25 ng/ml concentration, even at 50% recovery. The sensitivity of the present s-dot ELISA is 5 ng/ml, which is well ahead of the aforementioned yield. Hence, the current s-dot ELISA can be a valuable tool for routine qualitative analysis of cereal food samples.

## Conclusion

The developed s-dot ELISA is sensitive and can be used directly onto the field samples for routine analysis of OTA contamination. The present method developed in this study may supplement conventional mycotoxin detection techniques with respect to ease of performance and cost.

## Conflict of Interest Statement

The authors declare that the research was conducted in the absence of any commercial or financial relationships that could be construed as a potential conflict of interest.
