# Corrigendum: Development of sandwich dot-ELISA for specific detection of Ochratoxin A and its application on to contaminated cereal grains originating from India

**DOI:** 10.3389/fmicb.2023.1194327

**Published:** 2023-10-17

**Authors:** M. Venkataramana, R. Rashmi, Siva R. Uppalapati, S. Chandranayaka, K. Balakrishna, M. Radhika, Vijai K. Gupta, H. V. Batra

**Affiliations:** ^1^Division of Toxicology and Immunology, DRDO-BU Center for Life Sciences, Bharathiar University, Coimbatore, India; ^2^Microbiology Division, Defence Food Research Laboratory, Mysore, India; ^3^Department of Studies in Biotechnology, University of Mysore, Mysore, India; ^4^Discipline of Biochemistry, School of Natural Sciences, National University of Ireland Galway, Galway, Ireland

**Keywords:** Ochratoxin A, ELISA, monoclonal antibodies, HPLC, cereal grains

In the published article, there was an error in Figure 6. The original version of this Article contained the unintentional duplication of seven dot-blots out of a hundred in Figure 6.

The corrected Figure 6 and its caption “Detection of OTA positive fungal cultures by s-dot ELISA” appear below.

**Figure 6:**
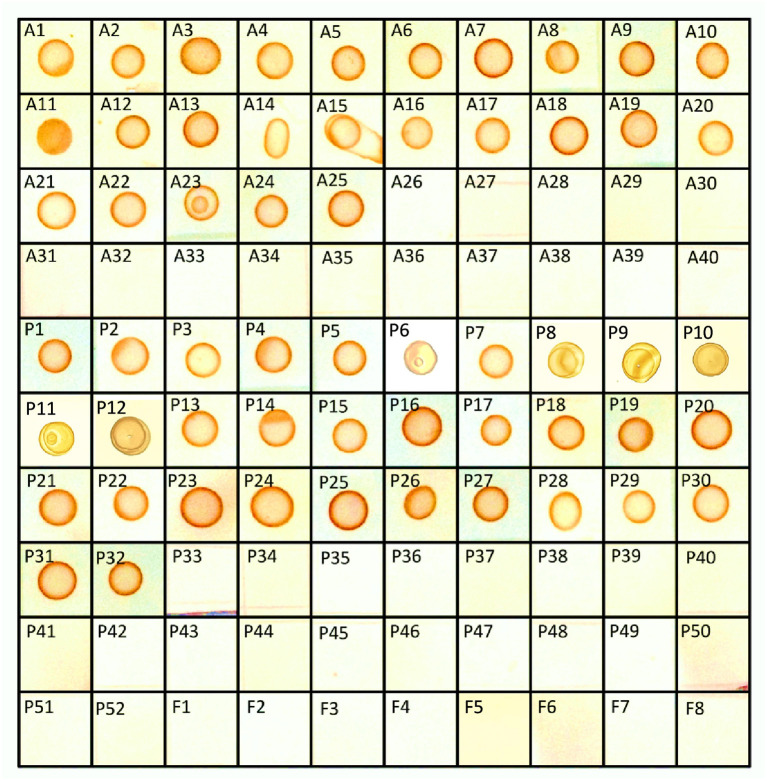
Detection of OTA positive fungal cultures by s-dot ELISA.

The authors apologize for this error and state that this does not change the scientific conclusions of the article in any way. The original article has been updated.

